# Treatment in Cholera

**Published:** 1843-11

**Authors:** 


*Treatment in Cholera.*—A physician of Freienwalde has, it is said, in the "Medic. Zeitung," proved the acetate of lead, with strychnine, to be effectual in causing the immediate cessation of the vomiting in sporadic cholera, and in tending to the speedy cure of that disease. The urinary secretion is, however, suspended under its emplayment, sometimes for as long a time as two days. Dr. Steinbach, of Bradenburg, is an advocate for the acetate in the same disease, but in combination with a solution of pure tannin. This mixture, he says, is specially indicated in the cases in which a softening of the gastro-intestinal mucous membrane is present.—*Ibid.*

